# Artificial intelligence-assisted reinterpretation of preclinical progeria research suggests a hierarchical nuclear-vascular resilience framework with translational implications

**DOI:** 10.7150/ijbs.133754

**Published:** 2026-08-21

**Authors:** Paolo Madeddu, Monica Cattaneo, Annibale Alessandro Puca

**Affiliations:** 1 Experimental Cardiovascular Medicine, Bristol Heart Institute, University of Bristol, Bristol, U.K.; 2 Cardiovascular Department, IRCCS MultiMedica, Milan, Italy.; 3 Department of Medicine and Surgery 'Scuola Medica Salernitana', University of Salerno, Fisciano, Italy.

**Keywords:** Hutchinson-Gilford progeria syndrome, cardiovascular aging, artificial intelligence-assisted reinterpretation, human-AI analytical dialogue, translational medicine

## Abstract

Preclinical research traditionally advances through hypothesis-driven experimentation that establishes mechanistic pathways to support translational development. While this approach has generated major biological insights, it may underemphasize alternative organizational patterns embedded within complex datasets, particularly in rare diseases where opportunities for experimental reiteration are limited. Recent advances in conversational artificial intelligence (AI) provide an opportunity to support structured analytical dialogue as a complementary approach for re-examining validated experimental observations.

Here, we evaluated the feasibility and informative value of an investigator-led structured analytical dialogue to reinterpret a previously published preclinical study of Hutchinson-Gilford Progeria Syndrome (HGPS), a rare disorder characterized by accelerated cardiovascular aging. Investigators defined the analytical questions, established interpretative boundaries, and critically evaluated successive AI-generated outputs, while the AI platform functioned exclusively as an analytical support tool for exploring complementary conceptual organization of experimentally validated findings. The original study showed that delivery of the longevity-associated *LAV-BPIFB4* gene preserved left ventricular diastolic function, reduced perivascular fibrosis, increased coronary arteriole density, and attenuated cellular senescence without modifying progerin accumulation.

Structured analytical dialogue generated complementary hierarchical interpretations of these observations. By integrating graphical dispersion with individual-level numerical data, the investigator-led dialogue identified heterogeneous response trajectories and suggested that cardiovascular protection may be viewed as emerging from coordinated interactions between nuclear stress adaptation and vascular remodelling within a broader resilience framework. These interpretations are presented as hypothesis-generating conceptual extensions rather than new experimental findings.

This study demonstrates the feasibility of structured investigator-led analytical dialogue as a complementary methodological approach for broadening interpretation of existing preclinical datasets while preserving the original experimental evidence. By making analytical reasoning more transparent and explicitly distinguishing validated observations from conceptual reinterpretation, this framework may assist prioritization of future mechanistic investigations, particularly in rare cardiovascular diseases where maximizing insight from existing datasets is especially important.

## Introduction

Biological research has traditionally progressed through a cognitive process in which theory informs hypothesis generation, experiments test cause-and-effect relationships, and mechanistic hierarchies are validated through targeted genetic or molecular perturbations, including transcriptional and post-transcriptional modulation. This framework has enabled major advances in biomedical science by providing a rigorous basis for establishing causal mechanisms and translating experimental discoveries into therapeutic strategies. Nevertheless, because hypothesis-driven investigation is inherently guided by predefined conceptual models, alternative biological relationships may remain underexplored 1. Re-examining validated experimental observations from alternative conceptual perspectives may generate complementary biological hypotheses and identify organizational patterns that were not emphasized in the original analysis, thereby helping to prioritize subsequent experimental investigation.

Artificial intelligence (AI) has increasingly contributed to translational cardiovascular medicine through applications such as imaging interpretation, predictive modelling, risk stratification, and automated signal assessment 2-5. The recent availability of conversational large language models (LLMs), capable of generating responses to free-text queries without task-specific programming, has created new opportunities for interactive analytical dialogue between investigators and AI 6. Their broad applicability has stimulated growing interest in clinical practice, medical education, and scientific research, while also raising important questions regarding reliability and the need for appropriate human oversight 7-9. The use of LLM platforms may be particularly valuable for advancing translational and clinical research on rare genetic diseases, where limited cohort sizes amplify the importance of extracting maximal information from existing datasets 10.

Hutchinson-Gilford Progeria syndrome (HGPS), caused by a mutation in the *LMNA* gene leading to progerin accumulation, is characterized by accelerated vascular aging, progressive arterial stiffening, and premature cardiovascular mortality 11,12. Current therapies that reduce progerin production or accumulation modestly extend survival but do not prevent the progressive cardiovascular complications that ultimately drive early death 13. These limitations highlight the need for adjunct strategies targeting downstream vascular dysfunction and maladaptive cardiac remodelling.

Several gene variants enriched in long-lived human populations may represent biological counterparts to progeroid mutations because they are associated with enhanced stress resilience, vascular homeostasis, and resistance to cellular senescence. Within this framework, we proposed that horizontal transfer of a healthy longevity-associated gene might mitigate both physiological and progeroid cardiovascular senescence. A longevity-associated variant (LAV) of the *BPI fold containing family B member 4 (BPIFB4)* gene has been linked to exceptional human healthspan and cardiovascular protection 14, and its viral vector-mediated delivery attenuated cardiovascular aging in aged mice 15. Building on this rationale, we recently investigated the same strategy in a murine model of HGPS, where *LAV-BPIFB4* delivery preserved diastolic left ventricular function, reduced perivascular fibrosis, and attenuated senescence signalling without altering progerin accumulation 16. These findings supported an intervention-anchored interpretation in which *LAV-BPIFB4* delivery was associated with adaptive changes in cardiac microvasculature and ventricular relaxation.

The present study examined the feasibility and informative value of a structured analytical dialogue in which investigators used AI to extend the interpretation of findings from the previously published HGPS study 16. Through qualitative assessment of graphical data distributions and cross-examination of individual-level numerical data, we explored whether the observations could be reorganized to generate additional mechanistic and translational hypotheses.

## Methods

### AI reporting and conceptual framework

The analytical dialogue was conducted using ChatGPT (GPT-5.5; OpenAI, San Francisco, CA, USA) accessed through the ChatGPT web interface under continuous investigator supervision. The analytical workflow was applied after publication of the original study and therefore functioned as a post-publication analytical reinterpretation rather than as part of the original experimental design. The analytical sessions were performed between February and June, 2026. The model was used in its standard conversational configuration without domain-specific fine-tuning, external biomedical knowledge-base integration, or retrieval-augmented generation. Because the model is proprietary and may evolve, reproducibility was supported by documenting representative prompts, analytical workflow, decision criteria, and reconstructed dialogue in **Supplementary Data 1**, rather than by relying solely on model version identification.

### Investigator-AI dialogue, boundaries, and responsibilities

The open-access PDF version of the published article from the journal website was uploaded on the ChatGPT platform. Individual-level numerical data, including echocardiography and histology endpoints from the *in vivo* study on HGPS mice and controls, and results from cell biology experiments, were supplied as dispersion images and tables. No unpublished data, annotations or external datasets were provided.

Investigators retained full responsibility for defining the analytical questions, directing the dialogue, critically evaluating AI-derived interpretations, and determining the concepts to be incorporated into the final interpretative framework.

Core analytical questions included:

- Can validated phenotypic outcomes be reorganized into alternative hierarchical frameworks without contradicting the original conclusions?
- Do dispersion patterns and extreme datapoints suggest context-dependent responsiveness beyond group-level averages?
- Does the absence of linear scaling between cardiac BPIFB4 expression and outcome support a threshold model of pathway engagement?
- What are the implications of cardiac BPIFB4 levels reflecting both endogenous and transgenic protein?
- Can vascular remodelling and senescence attenuation by *LAV-BPIFB4* delivery be interpreted as upstream regulatory layers rather than solely downstream effects?
- What translational implications emerge if vascular architecture and nuclear stress adaptation are viewed as resilience nodes?

Analytical boundaries were explicitly defined before starting the study. AI functioned exclusively as an analytical support tool to explore alternative hierarchical organization of biological processes already supported by the published data, identify complementary conceptual entry points, and propose biologically plausible mechanistic interpretations. AI-generated interpretations were retained only when they were fully consistent with the published dataset, biologically plausible, and supported by the existing scientific literature. Generation of new experimental claims, probabilistic predictions, statistical analyses, or causal assertions extending beyond the original evidence was excluded. When alternative or conflicting interpretations emerged, investigators reformulated the analytical question and repeated the dialogue until the proposed interpretations could be reconciled with the predefined analytical boundaries or were discarded. Accepted interpretations were further examined through a continuous validation loop grounded in established knowledge of HGPS pathophysiology and relevant published literature.

### Bias mitigation and reduction of directional influence

Because investigator-defined prompts inherently introduce the possibility of interpretative bias, several procedural safeguards were incorporated into the analytical workflow. First, prompts were structured around predefined analytical questions rather than desired outcomes, encouraging evaluation of alternative hierarchical interpretations independent of the original intervention-anchored framework. Second, orthogonal conceptual exploration required reinterpretation of identical observations from distinct biological entry points, thereby introducing a deliberate counter-perspective designed to reduce anchoring effects. Third, a dialogue-verification step ensured that prompts were accurately reconstructed and interpreted, minimizing drift toward implicit investigator assumptions. Finally, integration of dispersion-focused analysis — including examination of extreme datapoints and individual-level variability — shifted interpretation away from mean-based narratives that can reinforce prior expectations. Collectively, these measures were intended to reduce directional influence and to maintain analytical transparency within an investigator-led framework.

### Diagrammatic representation and graphical implementation

Diagrams were manually developed using BioRender (Toronto, Canada). The structured analytical dialogue informed their conceptual organization, whereas graphical implementation remained entirely under investigator control. Completed figures were subsequently reviewed during the analytical dialogue with the AI interface to confirm consistency with the evolving interpretative framework.

### Criteria ensuring methodological transparency and reproducibility

**Figure 1:**
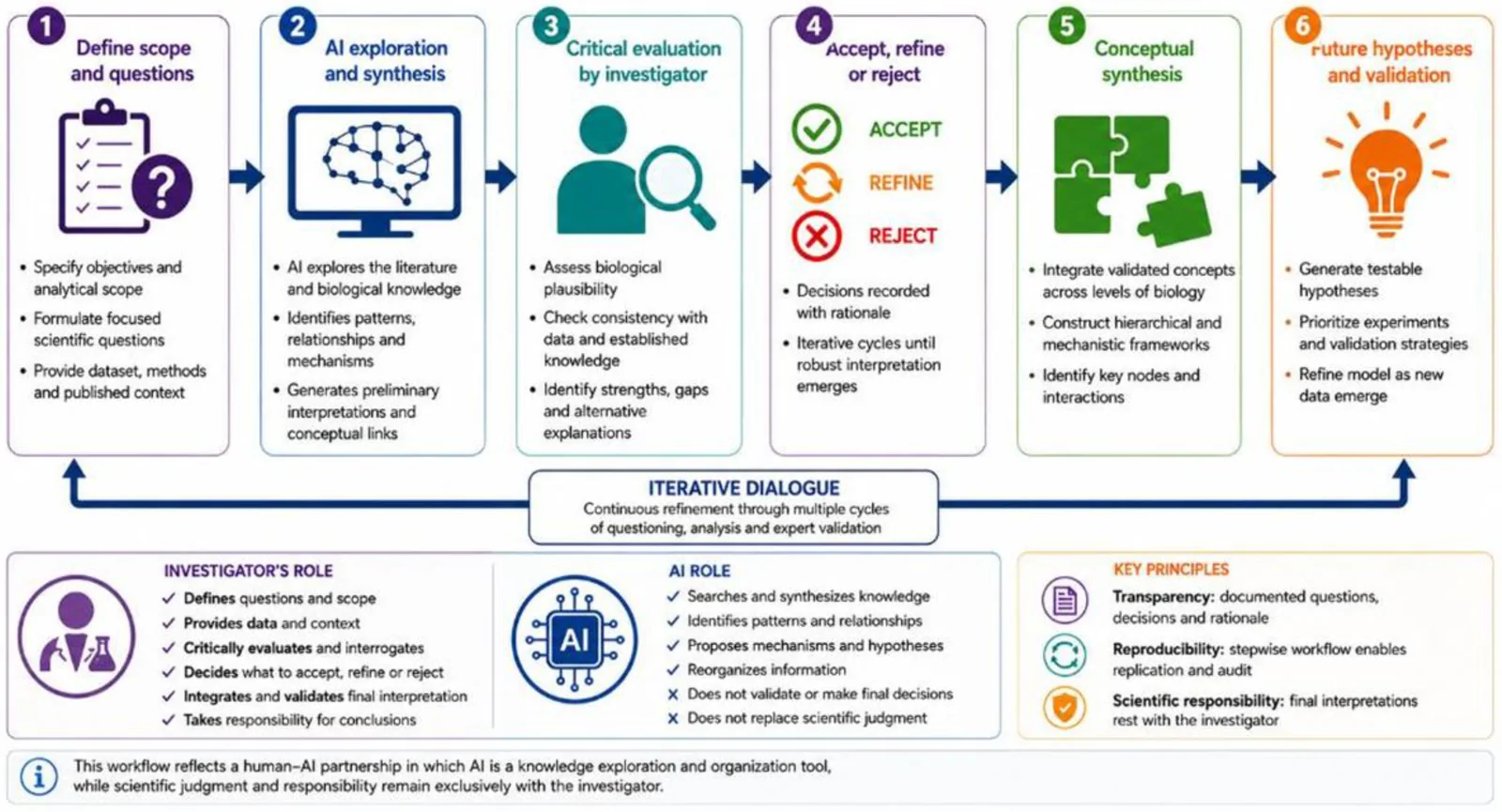
Structured workflow for investigator-led AI-assisted reinterpretation of experimental datasets. The workflow illustrates the analytical strategy adopted in this study. Investigators defined the biological questions, selected the published experimental data, critically evaluated successive AI-assisted outputs, and retained only interpretations consistent with the validated observations and established biological knowledge. The AI platform functioned exclusively as an analytical partner by reorganizing experimentally validated findings, exploring complementary conceptual relationships, and generating hypotheses for future investigation. The final conceptual synthesis resulted from iterative dialogue and continuous investigator supervision rather than autonomous AI inference.

**Supplementary Data 1** provides the analytical workflow, glossary of methodological terminology, representative investigator prompts, decision criteria, and a reconstructed analytical transcript documenting the evolution of the investigator-AI dialogue. In addition, **Figure 1** summarizes the investigator-led analytical workflow.

## Results

### Previously published experimental observations

The previously published study subjected to structured analytical reinterpretation investigated whether *LAV-BPIFB4* gene transfer could attenuate early manifestations of cardiomyopathy in a transgenic mouse model of HGPS 16.

As previously reported, therapeutic gene delivery increased BPIFB4 protein levels in the heart. Because the antibody used for protein detection does not distinguish murine from human BPIFB4, the measured increase reflected total cardiac BPIFB4 protein. This molecular change was associated with preserved left ventricular diastolic function, reduced perivascular fibrosis, increased coronary arteriole density, and reduced markers of cellular senescence.

Parallel experiments performed in fibroblasts derived from patients with HGPS showed that LAV-BPIFB4 transduction attenuated senescence and profibrotic signalling without modifying progerin levels. Transgene delivery reduced the expression of p53 and p21, crucial proteins in the stress response activated by progerin accumulation 17, and decreased the ability of HGPS fibroblasts to secrete epiregulin (EREG), a long-acting ligand of epidermal growth factor receptor (EGFR) implicated in profibrotic remodelling 18. In line with the *in vitro* data, *LAV-BPIFB4* delivery reduced EREG levels in the heart of HGPS mice.

These experimental observations supported an intervention-anchored framework in which *LAV-BPIFB4* delivery constituted the initiating event in a linear cause-effect cascade downstream to progerin. Improvements in microvascular architecture and extracellular matrix composition were interpreted as intermediate structural mediators linking molecular pathway activation to improved left ventricular relaxation. However, the original interpretation did not explicitly examine alternative hierarchical organization among the validated observations.

### AI-assisted reasoning provides complementary conceptual trajectories

AI-assisted analysis suggested that identical phenotypic outcomes could be organized into complementary hierarchical trajectories depending on the analytical entry point.

**Figure 2:**
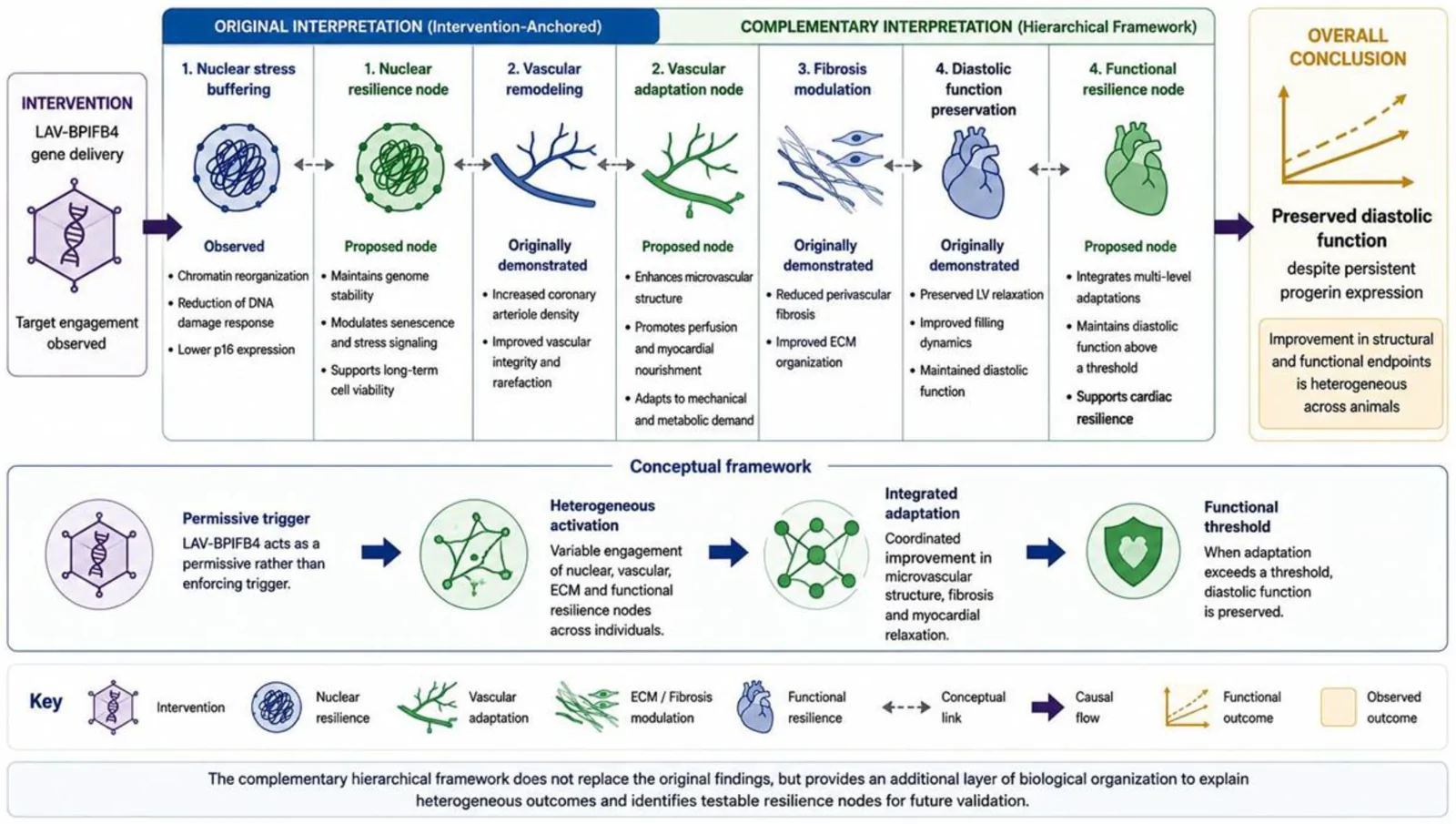
Figure 2. Comparison between the original intervention-anchored interpretation and the complementary hierarchical framework emerging from structured investigator-led analytical dialogue. The blue pathway summarizes the original mechanistic interpretation emphasizing attenuation of nuclear stress and cellular senescence, whereas the green pathway illustrates the complementary hierarchical organization emerging from the structured analytical dialogue, in which vascular remodeling and extracellular matrix adaptation are repositioned as candidate upstream determinants of functional improvement. Solid boxes and arrows indicate experimentally validated observations or established biological relationships. The figure illustrates how identical experimental observations can be reorganized into alternative, non-mutually exclusive conceptual frameworks without modifying the original experimental findings.

As shown in **Figure 2,** the complementary hierarchical interpretation suggested that the increased arteriole density and reduced perivascular fibrosis are candidate upstream determinants of functional improvement. Moreover, the reduction in p21 and other senescence markers advised that, by restoring nuclear stress adaptation, LAV-BPIFB4 could dampen pro-fibrotic signalling and create conditions favorable for vascular remodelling.

The AI-assisted dialogue also suggested that the above-mentioned vascular-first and nuclear stress-senescence perspectives are complementary hierarchical emphases rather than competing mechanisms. *LAV-BPIFB4* may function as a resilience amplifier—buffering downstream consequences of progerin-induced stress and linking reduced senescence burden to improved vascular integrity and diastolic function. This complementary framework is intended to generate testable biological hypotheses while remaining fully consistent with the original experimental observations.

### Dispersion analysis and identification of candidate resilience nodes

Visual inspection of graphical distributions revealed heterogeneous responses across treated animals, with extreme data points clustering within functional and structural endpoints. Direct interrogation of tabulated numerical data further suggested that these extremes may reflect structured biological variability rather than artefacts of visualization.

**Figure 3:**
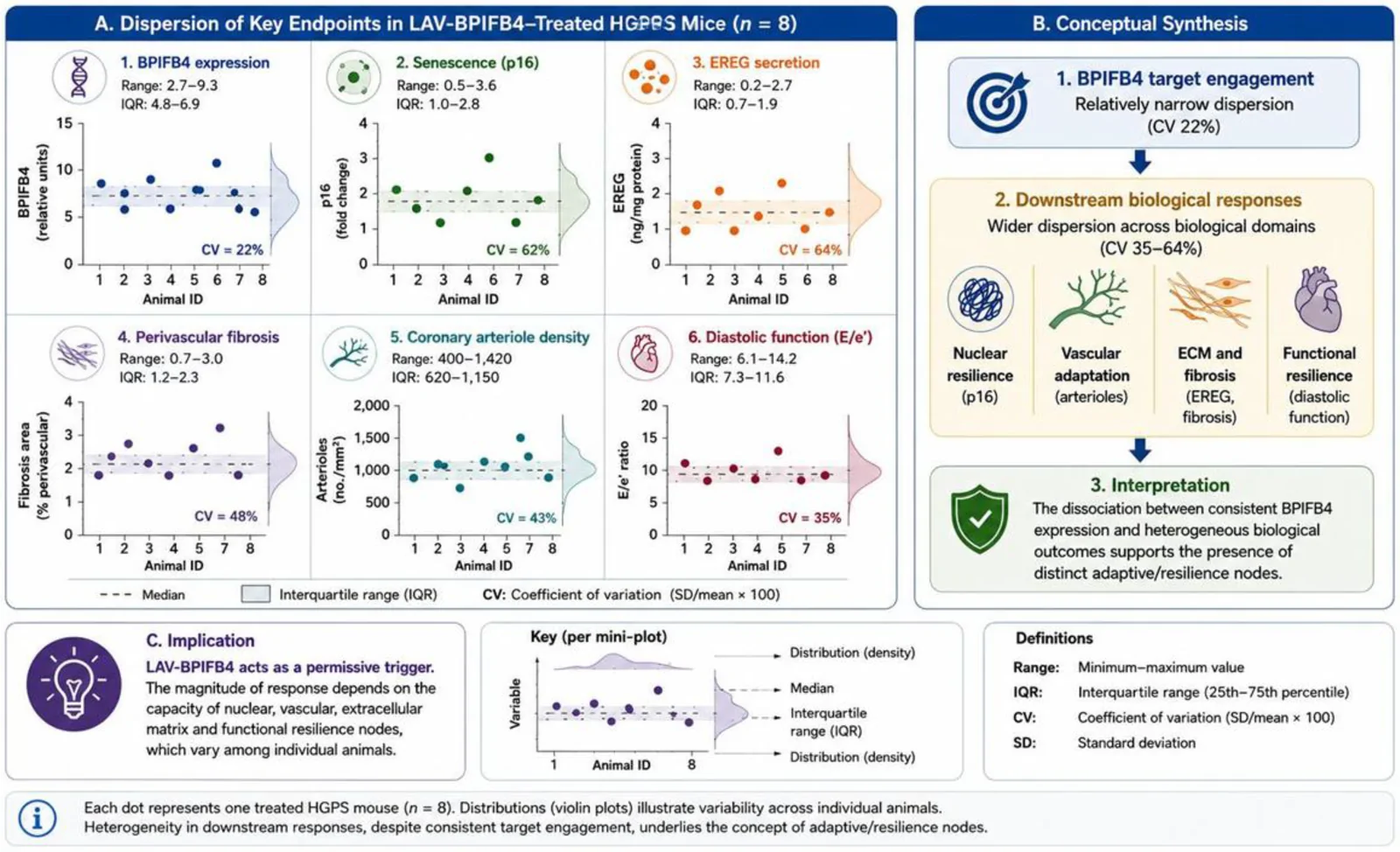
Variability-informed nuclear-vascular resilience framework emerging from structured analytical reinterpretation. Dispersion analysis showed relatively consistent cardiac BPIFB4 target engagement following gene delivery despite substantially greater variability in structural and functional responses. This dissociation suggested that LAV-BPIFB4 may function primarily as a permissive trigger enabling coordinated biological adaptation rather than as a quantitative determinant of therapeutic response. Integration of variability patterns identified four candidate resilience nodes—nuclear stress-senescence adaptation, vascular remodeling, extracellular matrix stiffness, and diastolic performance threshold—that may influence the magnitude of functional improvement. Together, these observations support a variability-informed conceptual framework in which preservation of cardiac function emerges from coordinated interactions among multiple adaptive mechanisms rather than from a simple linear relationship between transgene expression and biological outcome. The proposed resilience nodes represent hypothesis-generating conceptual interpretations that require prospective experimental validation.

The dialogue pinpointed that BPIFB4 expression increased consistently following treatment, indicating effective target engagement with relatively narrow dispersion compared with structural and functional variables. Notably, however, improvements in function and tissue remodelling did not scale linearly with BPIFB4 expression levels, suggesting that the modulation of BPIFB4 expression may function primarily as a permissive trigger rather than a quantitative driver. When examining the dispersion of the other validated data, the dialogue helped us to identify four specific nodes, as also illustrated in **Figure 3**:

- *Nuclear stress-adaptation senescence gating node.* Dispersion in baseline p16 levels suggested heterogeneity in nuclear stress burden and stress-response activation. The animals exhibiting greater senescence signalling appeared to derive larger therapeutic benefit.
- *Vascular remodelling and arteriole adaptation node.* Variability in arteriole density and vascular pericyte coverage paralleled a reduction in perivascular fibrosis.
- *Extracellular matrix stiffness node.* Animals with higher baseline perivascular fibrosis appeared to exhibit disproportionately large improvements.
- *Diastolic performance threshold node.* Functional improvement appeared to emerge when fibrosis and senescence markers crossed specific ranges.

Taken together, the identified nodes suggest a reorganized hierarchical framework in which cardiovascular protection emerges from coordinated interactions between nuclear stress adaptation and vascular structural remodelling rather than from a single linear molecular cascade.

## Discussion

This study illustrates the feasibility and informative value of using a structured analytical dialogue to extend interpretation of a previously published preclinical dataset from conceptual perspectives that were not emphasized in the original hypothesis-driven analysis. Rather than generating new biological evidence, the analytical process provided a transparent framework through which investigators re-examined validated experimental observations and developed complementary hypotheses for future investigation.

Applied to our previously published dataset, the structured analytical dialogue reaffirmed the primary biological signal associated with the intervention while suggesting a complementary hierarchical framework in which nuclear stress adaptation and vascular resilience may represent interacting regulatory processes associated with preservation of left ventricular diastolic function in progeria mice treated with a longevity-associated gene.

Our previous studies in non-progeric models of cardiomyopathy provide convergent evidence that *LAV-BPIFB4* gene therapy exerts both direct myocardial effects and broader vascular and stress-adaptive mechanisms. In type 2 diabetic mice, *LAV-BPIFB4* improved left ventricular relaxation, accompanied by adaptive reprogramming of contractile proteins 19,20. Mechanistic insight was reinforced in human induced pluripotent stem cell-derived cardiomyocytes, where exposure to recombinant LAV-BPIFB4 protein improved contractile performance 21. Additionally, delivery of the *LAV-BPIFB4* gene or recombinant protein rescued cardiac function and myocardial perfusion in diabetic and aged mice by improving microvascular density and pericyte coverage 22. *LAV-BPIFB4*-primed pericytes from failing human hearts exhibited reduced expression of senescence markers, increased stress resilience, and enhanced proangiogenic activity through a mechanism involving the nucleolar protein nucleolin 15. Taken together, these independent observations provide biological plausibility for the complementary interpretative framework generated in the present study, while remaining consistent with the original intervention-anchored mechanism. Importantly, they should not be regarded as independent validation of the hierarchical organization proposed through the structured analytical dialogue, which remains hypothesis-generating.

One contribution of the present analytical approach was to make explicit an interpretative process that investigators often perform implicitly when revisiting complex datasets. By systematically examining the published observations from alternative conceptual entry points while preserving the original experimental evidence, the structured analytical dialogue facilitated discussion of biological relationships that were not emphasized in the original report. In this respect, the added value of the analytical process lies not in replacing hypothesis-driven interpretation, but in complementing it through a transparent and reproducible framework for generating biologically grounded hypotheses.

A second contribution of the structured analytical dialogue was the systematic examination of variability within the published dataset. Rather than focusing exclusively on group-average responses, investigators combined qualitative assessment of graphical dispersion with interrogation of individual-level numerical data to explore whether heterogeneous treatment responses could provide additional biological insight. This exploratory analysis suggested that therapeutic responsiveness was not uniformly distributed across treated animals and prompted consideration of whether biological context might influence the magnitude of phenotypic benefit despite consistent target engagement.

Within this analytical framework, the dialogue generated the hypothesis that *LAV-BPIFB4* may function less as a linear dose-to-effect driver and more as an initiator of adaptive biological processes whose downstream consequences depend on the pre-existing cellular and vascular context. Accordingly, the apparent heterogeneity in treatment response was interpreted as a source of biological information rather than experimental noise. Because these observations were derived from exploratory reinterpretation of a single published dataset, they should not be interpreted as evidence for discrete responder and non-responder populations but rather as candidate response trajectories requiring prospective validation.

During the structured analytical dialogue, investigators also reconsidered the observation that increased BPIFB4 immunoreactivity in treated animals could reflect not only transgene expression but also endogenous BPIFB4. The dialogue suggested that this possibility was biologically plausible, given the reported involvement of BPIFB4 in stress-adaptive signalling pathways. Although the original dataset cannot distinguish between endogenous and transgene-derived protein, this complementary interpretation generated the hypothesis that therapeutic gene delivery might initiate adaptive signalling capable of reinforcing endogenous BPIFB4 expression. This hypothesis provides one possible explanation for the absence of an obvious linear relationship between measured BPIFB4 protein levels and downstream functional improvement but remains to be experimentally tested.

From this perspective, the analytical dialogue further suggested that the principal therapeutic objective may not necessarily be maximal BPIFB4 expression but sufficient target engagement to activate adaptive pathways. This interpretation generated the hypothesis that future studies could investigate the minimal level of target engagement associated with functional benefit and evaluate whether biological markers of vascular remodelling or senescence burden identify conditions under which treatment is most effective. Such investigations may ultimately contribute to optimization of dose selection and integration with complementary therapeutic strategies, including farnesyltransferase inhibition or emerging gene-modifying approaches; however, implications remain speculative and extend beyond the evidence provided by the present study.

One practical outcome of the structured analytical dialogue was the development of an illustrative roadmap for future investigation. When investigators asked the AI platform to help prioritize subsequent experimental steps within realistic resource constraints, the dialogue suggested an initial focus on defining the target-engagement window, followed by evaluation of candidate biomarkers of fibrosis and senescence, selective integration with standard therapies, and validation of complementary mechanistic hypotheses in human-relevant experimental systems. This roadmap is presented as an example of how structured investigator-led analytical dialogue may assist in prioritizing future studies rather than as a prescriptive development strategy. More generally, this approach may complement established forms of post-publication scientific discussion, such as editorials and commentaries, by providing a structured framework for re-examining published findings from alternative conceptual perspectives 22. This may be particularly valuable in rare genetic cardiovascular diseases, where small patient cohorts, limited availability of patient-derived material, and high experimental costs often restrict opportunities for experimental reiteration, making it especially important to maximize mechanistic insight from existing datasets.

An additional methodological consideration concerns our deliberate use of a general-purpose rather than a domain-adapted large language model. Domain-specific approaches, including fine-tuning on specialized biomedical corpora or integration of structured knowledge bases, may improve performance for narrowly defined analytical tasks. However, to our knowledge, no established domain-adapted AI platform has been specifically validated for HGPS datasets. Recent comparative studies indicate that such adaptation does not consistently outperform general-purpose language models on previously unseen biomedical reasoning tasks 23,24. Moreover, in a different application, a recent study of 121 rare diseases reported that a general-purpose conversational model achieved the highest diagnostic accuracy among the evaluated systems without disease-specific adaptation 25. Although diagnostic reasoning differs from investigator-led reinterpretation of experimental datasets, these findings suggest that broad reasoning capability does not necessarily depend on domain-specific adaptation. Accordingly, our objective was not to maximize disease-specific knowledge retrieval, but to evaluate whether a widely accessible conversational AI could support structured analytical dialogue under continuous investigator supervision. We considered this approach to be more appropriate for assessing the feasibility and generalizability of the proposed methodology. Whether domain-specific adaptation would further enhance—or modify—the generation of complementary biological hypotheses in studies such as ours remains an open question that warrants direct comparative investigation.

## Limitations

Several limitations should be acknowledged. First, this work represents a methodological proof-of-concept based on reinterpretation of a single previously published preclinical dataset. Consequently, the complementary conceptual framework generated through the structured analytical dialogue should not be regarded as independent biological validation of the proposed hierarchical organization**.** Dispersion-based interpretation retains an element of subjectivity despite integration with individual-level numerical analysis, and the proposed response trajectories, threshold-engagement model, and candidate resilience nodes remain analytical hypotheses requiring prospective evaluation in independent datasets.

Second, the analytical process relied on investigator-defined questions and continuous biological oversight. Although procedural safeguards—including predefined analytical boundaries, iterative evaluation, orthogonal conceptual exploration, and explicit acceptance criteria—were incorporated to reduce directional influence, alternative investigator groups might generate different complementary interpretations from the same dataset. This characteristic reflects the exploratory nature of structured analytical dialogue rather than a limitation unique to AI-assisted reasoning.

Finally, the present study was not designed to determine whether the proposed conceptual framework is biologically correct. Rather, its objective was to evaluate whether structured analytical dialogue could provide complementary perspectives that assist investigators in identifying hypotheses deserving future experimental investigation.

## Conclusions and Perspectives

This study illustrates the feasibility and informative value of incorporating structured analytical dialogue into investigator-led reinterpretation of experimental datasets. Rather than replacing hypothesis-driven investigation, the analytical process provided a transparent and reproducible framework through which validated observations could be re-examined from complementary conceptual perspectives while preserving the original experimental evidence.

Applied to a murine model of HGPS, the structured analytical dialogue generated complementary hypotheses suggesting that nuclear stress adaptation and vascular remodelling may represent interacting biological processes influencing therapeutic responsiveness following *LAV-BPIFB4* gene delivery. Future work should determine the reproducibility of this analytical strategy across independent datasets, biological systems, and investigator teams.

## Supplementary Material

Appendix and Supplementary figures and tables.
