# Supplementary material for: Artificial intelligence-assisted reinterpretation of preclinical progeria research suggests a hierarchical nuclear-vascular resilience framework with translational implications
Source: Int J Biol Sci. 2026 Aug 21;22(13):7349–57. doi: 10.7150/ijbs.133754 (PMC13540685; doi:10.7150/ijbs.133754)
Supplement: Supplementary file 1 [file ijbsv22p7349s1.pdf]

Appendix

Structured Investigator–AI Analytical Dialogue Underlying Post-publication Reinterpretation

Model used: ChatGPT (GPT-5.5, OpenAI).
Interaction period: [February-June 2026].
Mode: Investigator-led iterative analytical dialogue.

1. Purpose of this Supplement

This Supplement has been prepared in response to the reviewers' request for greater transparency regarding the analytical process underlying the AI-assisted reinterpretation presented in the manuscript. Because the principal contribution of the study is methodological rather than computational, the investigator–AI dialogue itself constitutes an essential component of the scientific record.

The objective of this document is therefore to provide a transparent account of how the reinterpretation evolved, from the initial methodological questions through successive stages of biological analysis to the final conceptual framework incorporated into the revised manuscript. The Supplement illustrates the progression of analytical reasoning, the iterative refinement of ideas, and the continuous investigator oversight that governed acceptance, modification, or rejection of AI-assisted interpretations.

The dialogue should not be interpreted as a source of new experimental evidence. Rather, it shows how structured interaction between investigators and an AI platform was used to reorganize previously validated observations into complementary conceptual frameworks while remaining fully consistent with the original experimental findings.

2. Analytical Workflow Summary

The analytical dialogue was not conducted according to a predefined script but evolved iteratively as new biological and methodological questions emerged. Retrospective examination of the complete interaction nevertheless revealed a reproducible analytical workflow consisting of sequential reasoning cycles. This workflow progressively matured during the dialogue and subsequently guided revision of the manuscript.

Each analytical cycle consisted of five consecutive steps:

1. **Investigator-defined analytical question.** A biological or methodological question arising from critical re-examination of the published study was formulated by the investigators.
2. **AI-assisted analytical exploration.** The AI generated one or more conceptual interpretations or alternative biological organizations while remaining anchored to the information provided by the investigators and established scientific knowledge.
3. **Critical investigator evaluation.** Each response was examined for consistency with the published experimental observations, biological plausibility, and potential translational relevance.
4. **Iterative refinement.** Interpretations considered insufficiently supported, overly speculative, or inconsistent with the available evidence were modified, challenged through additional dialogue, or rejected.
5. **Conceptual synthesis.** Accepted concepts were incorporated into the evolving analytical framework and subsequently translated into the manuscript.

The dialogue evolved through seven broad analytical phases:

Phase	Principal objective
**1**	Define the methodological rationale for AI-assisted reinterpretation
**2**	Develop orthogonal analytical strategies complementary to the original intervention-anchored framework
**3**	Explore alternative biological hierarchies, including vascular remodelling and nuclear stress adaptation
**4**	Re-examine biological variability through dispersion analysis, responder heterogeneity, candidate resilience nodes, and threshold-like pathway engagement
**5**	Reinterpret BPIFB4 protein expression and distinguish target engagement from adaptive pathway activation
**6**	Reflect on investigator–AI interaction, analytical bias, and methodological implications
**7**	Translate the analytical dialogue into a scientifically rigorous manuscript while distinguishing observed findings from conceptual synthesis and future hypotheses

Throughout the dialogue, AI-generated interpretations were accepted only when they:

- remained fully compatible with the published experimental observations;
- did not contradict established biological knowledge;
- provided additional explanatory or translational value beyond the original interpretation; and
- could be clearly distinguished from experimentally demonstrated findings.

Scientific responsibility remained entirely with the investigators, who generated the analytical questions, critically evaluated every response, requested clarification or revision where necessary, and determined which concepts were incorporated into the manuscript.

How this Supplement should be read

The dialogue documents the evolution of scientific reasoning rather than a sequence of definitive conclusions. Early chapters illustrate exploratory conceptual development and comparison of alternative analytical pathways. Later chapters show how these ideas were progressively refined, moderated, integrated, or rejected before incorporation into the manuscript. Accordingly, some concepts appear in a broader or more exploratory form within the dialogue than in the final manuscript, reflecting the natural evolution of the analytical process.

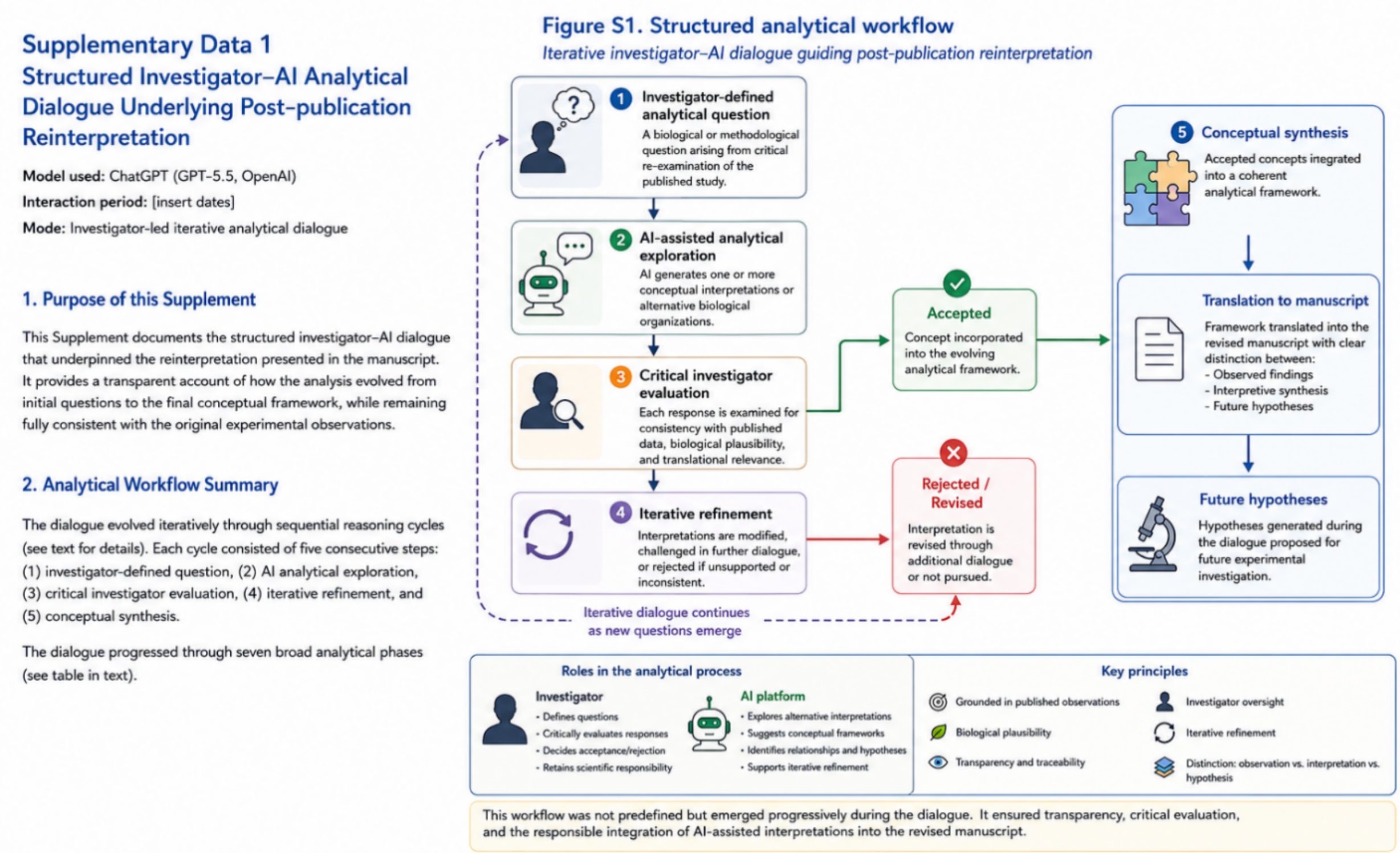

3. Explanatory Note

The following chapters present a **faithfully reconstructed analytical transcript** derived from the complete investigator–AI conversation. The reconstruction was prepared directly from the original dialogue and is intended to preserve the scientific reasoning process while improving readability and facilitating independent evaluation of the analytical workflow.

To enhance clarity, the dialogue has been organized into thematic chapters that reflect the chronological evolution of the investigation. Within each chapter, the sequence of investigator questions and AI responses has been preserved as closely as possible.

Minor editorial revisions were limited to correction of typographical errors, removal of duplicated exchanges, consolidation of repetitive prompts, and omission of discussions exclusively related to language editing, formatting, reference management, or other editorial aspects not directly relevant to the scientific reasoning. Where brief linking sentences were required to preserve continuity following removal of repetitive material, these have been explicitly identified as editorial notes.

No scientific concepts, analytical steps, or investigator decisions have been intentionally omitted. The progression of reasoning, the evolution of alternative conceptual frameworks, and the continuous investigator oversight faithfully reflect the original dialogue.

The chapters that follow therefore document not only the conclusions ultimately incorporated into the manuscript but also the analytical pathway through which competing ideas were explored, critically evaluated, refined, or discarded before the final conceptual framework was established.

4. Glossary of analytical terminology

The following definitions are provided to facilitate interpretation of the analytical dialogue and the revised manuscript. These terms are used in a methodological rather than experimental sense and describe conceptual constructs that emerged during the investigator–AI dialogue. Unless explicitly supported by

experimental evidence, the terms should be interpreted as analytical frameworks or hypothesis-generating concepts rather than validated biological mechanisms.

Intervention-anchored framework

An interpretative framework in which the therapeutic intervention is considered the initiating event, and downstream molecular, structural, and functional changes are interpreted as consequences of successful pathway engagement.

Orthogonal analysis

Examination of the same dataset from a complementary conceptual entry point independent of the original hypothesis. This approach explores alternative biological organization while remaining compatible with the experimentally supported interpretation.

Resilience nodes

Interdependent biological processes proposed through AI-assisted reinterpretation to contribute to tissue adaptation and functional stability under stress. In the present study, nuclear stress adaptation and vascular remodelling are presented as **candidate** resilience nodes requiring prospective experimental validation.

Responder heterogeneity

Variation in treatment response among individuals within the same experimental group, interpreted as potentially reflecting biological context in addition to experimental variability. Within the present study, this concept is proposed as a hypothesis-generating interpretation derived from exploratory analysis of data dispersion.

Structured investigator–AI dialogue

An investigator-led iterative analytical process in which successive scientific questions, AI-assisted conceptual interpretations, critical biological evaluation, and repeated refinement are used to explore complementary organization of an existing dataset in a transparent and reproducible manner.

Threshold engagement model

A hypothesis-generating framework proposing that therapeutic benefit may emerge once sufficient biological pathway engagement has been achieved rather than increasing linearly with the magnitude of target expression.

Vascular–nuclear resilience perspective

An interpretative perspective in which vascular remodelling and nuclear stress adaptation are considered candidate upstream organizational layers contributing to preservation of cardiac function while maintaining the experimentally validated intervention-anchored interpretation.

Vertical reasoning

Traditional hypothesis-driven reasoning that progresses from hypothesis formulation through experimental testing toward mechanistic validation using targeted biological perturbation.

Chapter 1. Origin of the analytical dialogue

Defining the methodological question

Investigator

The purpose of this dialogue is not to generate new experimental data but to determine whether artificial intelligence can help reinterpret an already published preclinical study.

The original work had established a clear intervention–outcome relationship supported by experimental evidence. However, publication does not necessarily exhaust the scientific value of a dataset. During translational development, investigators frequently return to published results to generate new hypotheses, reassess mechanistic priorities, identify biomarkers, refine therapeutic strategies, and design subsequent experiments.

Can AI assist this post-publication analytical process in a structured and transparent way?

AI

Yes.

The informative life of an experimental dataset generally extends well beyond publication. Published results continue to influence scientific reasoning throughout the translational pathway by supporting mechanistic refinement, guiding experimental design, prioritizing biomarkers, informing dose optimization, and identifying opportunities for clinical development.

Traditionally, this post-publication reinterpretation relies almost entirely on investigator experience and iterative discussion within research groups.

Artificial intelligence may contribute differently.

Rather than replacing scientific expertise or generating new biological evidence, AI can function as a structured reasoning interface capable of systematically exploring alternative conceptual organizations while remaining anchored to the validated experimental observations.

In this context, the objective is not discovery of new data but expansion of the conceptual space available for interpreting existing evidence.

Investigator

This distinction is important.

The manuscript should therefore avoid presenting AI as a hypothesis generator or autonomous decision-maker.

Instead, AI should function as a reflective analytical companion supporting investigator-led reasoning.

AI

Agreed.

The analytical workflow should preserve a clear hierarchy of responsibilities.

The investigator defines the biological problem, formulates the analytical questions, evaluates biological plausibility, accepts or rejects proposed interpretations, and remains fully responsible for the scientific conclusions.

The AI contributes by proposing alternative conceptual organizations, highlighting relationships that may not be immediately apparent, and stimulating critical examination of established interpretative frameworks.

Scientific authority therefore remains entirely with the investigator.

Why select a published study in Hutchinson–Gilford Progeria Syndrome?

Investigator

If this methodology is to be evaluated, the choice of dataset becomes important.

Why is our published BPIFB4 study in Hutchinson–Gilford Progeria Syndrome an appropriate model?

AI

Several characteristics make this study particularly suitable.

First, the original publication already established a coherent biological narrative supported by experimental evidence. This provides a stable evidentiary baseline against which reinterpretation can be assessed.

Second, the intervention improved cardiovascular phenotype without modifying progerin accumulation. This creates an opportunity to explore whether downstream adaptive mechanisms may contribute to therapeutic benefit despite persistence of the primary genetic defect.

Third, rare diseases inherently generate relatively small datasets, limiting opportunities for repeated experimentation. Consequently, maximizing the analytical value of existing data has particular translational importance.

Finally, the study integrates molecular, structural, vascular, and functional endpoints, allowing alternative biological hierarchies to be explored without introducing new experimental observations.

Investigator

Therefore, the purpose is not to question the original publication.

Rather, the objective is to determine whether the same validated observations can support complementary conceptual frameworks capable of extending translational interpretation.

AI

Correct.

The analytical dialogue should preserve three fundamental principles throughout:

1. Experimental observations remain unchanged.
2. Original biological conclusions remain valid.
3. Alternative interpretations must remain compatible with both the published evidence and established biological knowledge.

The dialogue therefore seeks conceptual expansion rather than reinterpretation through contradiction.

Transition toward analytical exploration

Investigator

How should the analytical process begin?

AI

The dialogue should first establish the conventional intervention-anchored interpretation of the published study.

Only after defining this reference framework should alternative conceptual organizations be explored.

This sequence provides an explicit comparator throughout the analysis and prevents reinterpretation from becoming detached from the original experimental evidence.

Consequently, the next analytical stage should examine whether the validated observations can be reorganized through orthogonal conceptual entry points while preserving their experimental meaning.

Editorial note

The analytical considerations developed in this opening dialogue formed the basis for the methodological framework described in the Introduction and Methods sections of the revised manuscript. They established the central premise that structured human–AI dialogue was intended to expand conceptual interpretation while preserving the integrity of the original experimental evidence.

Chapter 2. From conventional hypothesis-driven interpretation to orthogonal analytical reasoning

Introducing an alternative analytical perspective

Investigator

Biological research is usually conducted through a vertical reasoning process. Investigators formulate a hypothesis, design experiments to test cause-and-effect relationships, and subsequently validate the proposed mechanism using genetic or pharmacological perturbation.

Can an already published dataset be examined from a different conceptual entry point without challenging the original conclusions?

AI

Yes.

Conventional biological reasoning follows a predominantly vertical structure.

Theory generates hypotheses.

Hypotheses guide experiments.

Experimental observations establish causal relationships.

Mechanistic studies then strengthen or refute the proposed pathway.

This strategy is fundamental for demonstrating biological causality.

However, once a dataset has been generated and experimentally validated, the same observations may still permit alternative conceptual organizations.

Rather than testing a new hypothesis experimentally, investigators may ask whether the validated findings can be reorganized into complementary mechanistic hierarchies that were not emphasized during the original interpretation.

This represents an orthogonal analytical perspective rather than a competing biological theory.

Investigator

Therefore, orthogonal reasoning should not replace conventional hypothesis-driven science.

AI

Correct.

The purpose of orthogonal reasoning is complementary rather than competitive.

It preserves the experimental evidence while exploring alternative biological entry points.

Consequently, the dialogue should remain firmly anchored to the published observations.

Every reinterpretation should satisfy three conditions:

- remain compatible with the experimental data;
- remain biologically plausible;
- provide additional explanatory or translational value.

Can multiple hierarchies coexist?

Investigator

If several conceptual organizations are biologically plausible, should one be considered correct and the others incorrect?

AI

Not necessarily.

Complex biological systems frequently permit multiple hierarchical organizations.

Experimental observations establish what occurred.

Hierarchy determines how these observations are conceptually organized.

Different hierarchies may therefore coexist without contradicting one another, provided that they remain compatible with the underlying evidence.

Consequently, reinterpretation concerns organization rather than replacement of biological mechanisms.

Investigator

Would this distinction help explain why different experts often interpret the same paper differently?

AI

Yes.

Investigators usually agree on the experimental observations.

Differences more commonly arise in the biological hierarchy assigned to those observations.

Experience, disciplinary background, and prevailing conceptual models influence which variables are considered primary and which are viewed as downstream consequences.

Structured dialogue allows these implicit hierarchies to become explicit and therefore open to critical evaluation.

Can AI contribute to this process?

Investigator

Many current applications of artificial intelligence focus on prediction, classification, or image analysis.

What role could AI play in this analytical setting?

AI

The role differs fundamentally.

AI is not being asked to predict experimental outcomes or generate new biological evidence.

Instead, it functions as a structured reasoning interface.

Its contribution consists of exploring alternative conceptual organizations that remain compatible with validated observations.

The objective is to widen the analytical search space while allowing the investigator to determine which interpretations remain biologically credible.

Accordingly, the dialogue becomes an iterative reasoning exercise rather than an automated discovery process.

Investigator

Can AI itself determine which interpretation is biologically correct?

AI

No.

The AI has no independent access to experimental truth.

Its responses are evaluated entirely by the investigator.

Interpretations inconsistent with published observations or established biological knowledge should be rejected.

Consequently, biological validity remains under investigator control throughout the analytical process.

Choosing the analytical entry points

Investigator

If we are going to reinterpret the published BPIFB4 study systematically, should we define the conceptual pathways before beginning the dialogue?

AI

Yes.

Predefining broad analytical domains improves transparency and reduces the risk of post hoc interpretation.

Rather than exploring unlimited possibilities, the dialogue can be organized around a small number of complementary conceptual trajectories.

Each trajectory should represent a distinct biological perspective while remaining compatible with the original experimental observations.

Investigator

Which trajectories appear most appropriate for the present study?

AI

Three analytical domains appear particularly suitable.

1. Intervention-anchored framework

The original biological interpretation, beginning with BPIFB4 delivery and following the established sequence of downstream molecular, structural and functional effects.

2. Nuclear stress pathway

Exploring whether adaptation to persistent nuclear stress may organize the observed reduction in senescence despite unchanged progerin.

3. Vascular-first systems pathway

Examining whether preservation of vascular architecture provides an upstream organizational layer capable of integrating extracellular matrix remodeling and ventricular function.

These pathways should initially be explored independently before assessing whether they converge into a unified conceptual framework.

Investigator

Therefore, the objective is not to identify the "correct" hierarchy but to determine whether complementary organizations emerge from the same experimental observations.

AI

Exactly.

The dialogue should preserve the original intervention-anchored interpretation while evaluating whether additional organizational structures become apparent through systematic orthogonal reasoning.

The analytical process therefore proceeds by comparison rather than replacement.

Editorial note

The concepts developed during this phase of the dialogue established the methodological basis for the investigator-defined analytical prompts subsequently incorporated into the structured workflow. They also motivated the organization of reinterpretation into predefined conceptual domains, which later evolved into the nuclear stress, vascular resilience, and translational analytical frameworks described in the manuscript.

Chapter 3. Exploring alternative biological organizations of the BPIFB4–HGPS dataset

Searching for complementary mechanistic frameworks

Investigator

The original publication interpreted the biological effects of LAV-BPIFB4 through a conventional intervention-anchored framework: gene delivery increased BPIFB4 expression, leading to structural and functional improvements. Can the same observations be reorganized into alternative biological hierarchies without contradicting the published conclusions?

AI

Yes.

The intervention-anchored framework remains entirely valid because it reflects the experimental design.

However, the same observations can also be organized from different biological entry points.

Rather than asking **"What does BPIFB4 do?"**, one may ask **"What biological processes best organize the observed phenotype?"**

This shifts attention from the intervention itself toward the adaptive responses that ultimately determine cardiac function.

Several complementary organizational pathways appear plausible.

First exploratory pathway: autophagy and TFEB

Investigator

Since progerin is known to impair proteostasis, could autophagy represent the principal organizing mechanism?

AI

This is biologically plausible.

Progerin accumulation disrupts nuclear architecture and impairs nucleocytoplasmic trafficking. Experimental studies have reported reduced activity of transcription factor EB (TFEB), a master regulator of lysosomal biogenesis, autophagy, and cellular quality-control mechanisms.

Restoration of TFEB-dependent pathways could theoretically reduce cellular stress, improve proteostasis, and attenuate senescence despite persistence of progerin.

However, the published BPIFB4 dataset contains no measurements of TFEB activity, autophagic flux, or lysosomal function.

Consequently, although biologically attractive, this pathway would require substantial extrapolation beyond the available evidence.

Investigator

Would this become the central interpretation?

AI

Probably not.

It represents a plausible mechanistic hypothesis but is weakly anchored to the measured variables.

It is better considered as a possible downstream explanatory pathway rather than the principal organizational framework.

Investigator decision

The TFEB/autophagy pathway was retained as a mechanistic hypothesis but not selected as the primary hierarchical organization.

Second exploratory pathway: vascular remodeling

Investigator

Could vascular remodeling provide a stronger organizing principle?

AI

The published observations support this possibility more directly.

The dataset documents increased coronary arteriole density together with reduced perivascular fibrosis and preserved ventricular relaxation.

Unlike TFEB, these variables were measured directly.

Microvascular integrity influences myocardial perfusion, extracellular matrix homeostasis, oxygen delivery, and ventricular compliance.

Consequently, vascular remodeling represents a biologically measurable adaptive process linking molecular changes to organ function.

Investigator

Would this interpretation contradict the original paper?

AI

No.

The intervention remains the initiating event.

Only the hierarchical emphasis changes.

Instead of considering vascular remodeling simply as a downstream consequence of BPIFB4 delivery, it becomes a major adaptive layer through which functional preservation may emerge.

Investigator decision

The vascular pathway was selected for further exploration.

Third exploratory pathway: nuclear stress adaptation

Investigator

One finding continues to intrigue me.

Progerin levels remained unchanged, yet senescence markers were reduced.

Could adaptation to nuclear stress represent another organizing principle?

AI

This possibility appears particularly interesting.

If progerin accumulation persists, the reduction in p21 and related senescence markers cannot reasonably be attributed to correction of the primary genetic defect.

Instead, the observations suggest increased tolerance of persistent nuclear injury.

The intervention therefore appears to buffer the downstream consequences of progerin rather than eliminating its cause.

This shifts the interpretation from mutation correction toward enhancement of cellular resilience.

Investigator

How does this differ from the vascular interpretation?

AI

The vascular pathway begins at tissue organization.

The nuclear stress pathway begins at the cellular response to persistent nuclear injury.

They therefore represent different analytical entry points rather than competing biological mechanisms.

One starts with the vasculature.

The other starts with cellular adaptation.

Both converge toward preservation of myocardial structure and function.

Comparing the candidate pathways

Investigator

Can we compare the relative strengths of these different interpretations?

AI

Yes.

Each pathway may be evaluated according to three criteria.

1. Direct support from the published dataset

- Intervention-anchored framework: very strong.
- Vascular remodeling: strong.
- Nuclear stress adaptation: moderate.
- TFEB/autophagy: indirect.

2. Biological plausibility

All four pathways are biologically plausible and consistent with current knowledge.

3. Translational value

The vascular and nuclear stress pathways appear particularly valuable because they integrate multiple measured variables and generate experimentally testable hypotheses for future work.

Investigator

Should only one framework be retained?

AI

Not necessarily.

The strongest interpretation may emerge from combining complementary pathways into a hierarchical network.

Rather than selecting one dominant mechanism, the dialogue suggests that intervention-anchored reasoning, vascular remodeling, and nuclear stress adaptation represent different levels of biological organization.

Investigator decision

The analytical strategy shifted from identifying a single alternative mechanism toward integrating complementary hierarchical frameworks.

This transition marked an important conceptual turning point in the dialogue.

Emergence of systems-level integration

Investigator

Instead of choosing between these pathways, could they represent different layers within the same adaptive network?

AI

Yes.

This appears to be the most coherent interpretation.

BPIFB4 delivery remains the initiating intervention.

Nuclear stress adaptation represents the cellular response.

Vascular remodeling represents the tissue-level adaptation.

Extracellular matrix remodeling links vascular integrity to myocardial mechanics.

These adaptive layers converge on preservation of ventricular relaxation.

Rather than replacing the original intervention-anchored interpretation, this systems-level organization expands it by introducing hierarchical interactions among experimentally observed variables.

Investigator

This appears closer to systems biology than to classical linear causality.

AI

Correct.

The dialogue has progressively shifted from a linear intervention–effect sequence toward a hierarchical adaptive network.

Importantly, this transition emerged from repeated comparison of alternative organizational pathways rather than from introduction of new experimental evidence.

Editorial note

This stage of the dialogue represented a major conceptual transition.

Instead of searching for a single alternative mechanism, the analytical process progressively converged on the idea that multiple biologically plausible entry points could coexist within a common hierarchical framework.

This reasoning subsequently informed the development of Figures 1 and 2, the Results section describing complementary conceptual trajectories, and the Discussion emphasizing interdependent nuclear and vascular adaptive layers.

Chapter 4. From average treatment effects to biological heterogeneity

Re-examining the information contained within the dataset

Investigator

The original publication interpreted the study using conventional group comparisons based on mean values and statistical significance. However, while reviewing the figures, I was struck by the apparent heterogeneity among individual animals. Could this variability itself contain biologically meaningful information?

AI

Potentially yes.

Group averages summarize the dominant biological effect and remain essential for demonstrating treatment efficacy. However, they also compress the variability of individual responses.

Visual inspection of individual datapoints can sometimes reveal patterns that are not apparent from summary statistics alone.

Rather than asking only whether treatment changes the mean response, one may ask why animals receiving the same intervention exhibit different degrees of structural or functional improvement.

This shifts the analytical focus from average efficacy toward biological responsiveness.

Investigator

Would this constitute a new statistical analysis?

AI

No.

The published statistical analysis remains unchanged.

Inspection of dispersion represents an exploratory analytical exercise intended to generate biological hypotheses rather than confirm them statistically.

Consequently, any interpretation arising from variability must be presented explicitly as hypothesis-generating.

Exploring structured variability

Investigator

Can the apparent dispersion simply reflect random experimental noise?

AI

That possibility cannot be excluded.

However, when technical artefacts have been minimized through appropriate experimental procedures, biological variability itself may become informative.

The important question is not whether variability exists—it invariably does—but whether the pattern of variability appears compatible with underlying biological organization.

The present dataset cannot establish this formally.

Nevertheless, the distribution of several variables appears sufficiently structured to justify further analytical exploration.

Investigator

Should we therefore regard the extreme datapoints as biologically important?

AI

Not automatically.

Extreme observations should not be interpreted as evidence of responder subgroups without independent validation.

However, they may identify biological conditions worthy of further investigation.

Accordingly, the dialogue should distinguish clearly between observation, interpretation, and hypothesis.

Relationship between target engagement and biological response

Investigator

One aspect particularly attracted my attention.

BPIFB4 expression appeared to increase consistently in treated animals, whereas fibrosis, arteriole density, and diastolic function exhibited considerably greater variability.

Could this discrepancy itself be biologically informative?

AI

Yes.

This comparison suggests that successful target engagement alone may not fully determine therapeutic outcome.

If expression changes relatively uniformly whereas downstream phenotypes remain heterogeneous, additional biological factors are likely to influence treatment response.

Consequently, BPIFB4 expression may function primarily as an initiating event, whereas the magnitude of benefit depends upon the adaptive capacity of the recipient tissue.

This interpretation remains entirely compatible with the published data while providing an alternative perspective on therapeutic responsiveness.

Investigator

Does this imply that expression is unimportant?

AI

No.

Effective expression remains necessary.

However, once adequate biological activity has been achieved, downstream adaptive processes may become the principal determinants of phenotypic variability.

Thus, target engagement and therapeutic response should not necessarily be viewed as quantitatively equivalent.

Emergence of candidate resilience nodes

Investigator

Can these heterogeneous responses be organized into broader biological categories?

AI

Rather than considering each variable independently, the observations appear to cluster into several interacting biological domains.

These domains may be regarded as candidate resilience nodes because each represents an adaptive process capable of influencing overall cardiovascular outcome.

The principal candidate nodes emerging from the dialogue are:

Nuclear stress adaptation

reflecting variability in senescence-associated signalling.

Microvascular structural adaptation

reflecting arteriole density and vascular organization.

Extracellular matrix remodelling

reflecting perivascular fibrosis and myocardial stiffness.

Functional adaptation

represented by preservation of ventricular relaxation.

These should be regarded as conceptual organizational units rather than experimentally validated mechanistic entities.

Investigator

Could these nodes explain why animals respond differently despite similar treatment?

AI

They provide one possible conceptual explanation.

If therapeutic benefit depends upon the interaction among several adaptive processes rather than a single molecular event, biological variability would be expected even under comparable levels of target engagement.

This interpretation naturally introduces the concept of biological resilience.

However, the present dataset is insufficient to establish causal relationships among these nodes.

Future studies would be required to determine their relative hierarchy.

Emergence of threshold thinking

Investigator

During our discussion, I have the impression that the response does not increase continuously with expression.

Rather, it seems to appear once a certain biological condition has been achieved.

AI

That impression is compatible with the observed patterns.

Instead of a continuously linear relationship, one may hypothesize that adaptive pathways require sufficient biological engagement before measurable tissue benefit becomes evident.

Beyond this point, additional increases in expression might produce progressively smaller incremental effects because downstream adaptive capacity becomes limiting.

Importantly, this represents a conceptual hypothesis rather than a demonstrated biological threshold.

Investigator

Would this interpretation have translational implications?

AI

Potentially.

If confirmed experimentally, future dose-optimization studies might focus on achieving reliable pathway engagement rather than maximal transgene expression.

However, the present study neither measures dose-response relationships nor validates such thresholds.

Accordingly, this implication should be presented cautiously as a possible consequence of the analytical reinterpretation.

Integrating heterogeneity into the analytical framework

Investigator

Our discussion has progressively moved away from average treatment effects toward variability itself.

Does this change the overall analytical philosophy?

AI

Yes.

The dialogue has evolved from asking whether BPIFB4 improves cardiac phenotype to asking why improvement varies among apparently similar animals.

This shift does not alter the published conclusions regarding efficacy.

Instead, it introduces biological heterogeneity as an additional analytical dimension.

Within this framework, variability is no longer viewed solely as statistical dispersion but also as a potential source of mechanistic insight.

Investigator

I think this represents one of the principal conceptual advances emerging from the dialogue.

AI

I agree.

The reinterpretation does not generate new biological observations.

Rather, it extracts additional conceptual information from existing observations by treating biological heterogeneity as an analytical opportunity rather than merely experimental noise.

Editorial note

This stage of the dialogue represented the transition from conventional interpretation based on average treatment effects to a systems-oriented perspective incorporating biological heterogeneity. The concepts of candidate resilience nodes, pathway engagement, threshold-like behaviour, and responder variability subsequently became central elements of the revised Results and Discussion. Throughout manuscript revision these concepts were consistently presented as analytical hypotheses requiring prospective experimental validation rather than as experimentally demonstrated mechanisms.

Chapter 5. Reinterpreting BPIFB4 expression: from transgene abundance to pathway engagement

A methodological observation revisited

Investigator

While revising the manuscript, I realized that one methodological aspect deserves further consideration.

In the original publication, cardiac BPIFB4 protein expression was assessed using an antibody that recognizes both endogenous murine BPIFB4 and the human BPIFB4 transgene. Consequently, the increase in signal observed after treatment cannot be interpreted as reflecting only expression of the transferred gene.

This limitation was already acknowledged in the original publication.

However, what could be the biological implications of this observation for reinterpreting the dataset?

AI

This question shifts the discussion from analytical methodology to biological interpretation.

The important point is not simply that the antibody lacks species specificity.

Rather, the measured BPIFB4 signal may represent two biologically distinct processes occurring simultaneously:

- successful expression of the therapeutic transgene;
- modulation of endogenous BPIFB4 protein within the recipient myocardium.

Accordingly, the measured increase in BPIFB4 should not necessarily be interpreted solely as a quantitative marker of gene transfer.

Instead, it may represent the overall biological response of the BPIFB4 pathway.

Investigator

This distinction was not considered in the original publication.

Would this change the biological interpretation?

AI

The original conclusions remain unchanged.

However, the biological meaning assigned to BPIFB4 expression becomes broader.

Instead of representing only transgene abundance, the measured protein level may indicate overall pathway engagement resulting from interaction between exogenous gene delivery and endogenous adaptive responses.

Consequently, expression becomes a marker of biological activation rather than simply vector performance.

Linking pathway engagement to biological variability

Investigator

This interpretation appears particularly interesting when considered together with the heterogeneous structural responses observed among treated animals.

Could these two observations be related?

AI

Potentially yes.

If BPIFB4 expression reflects successful activation of a biological programme rather than simply the amount of transferred gene, one would not necessarily expect a linear relationship between protein abundance and downstream phenotypic improvement.

Instead, once sufficient pathway engagement has been achieved, subsequent differences in therapeutic response may depend predominantly upon the biological condition of the recipient tissue.

Under this interpretation, variation in fibrosis regression, vascular remodelling, or ventricular relaxation reflects differences in tissue responsiveness rather than differences in transgene expression.

Investigator

Would this imply that expression is no longer important?

AI

No.

Effective expression remains essential because it initiates pathway activation.

The reinterpretation simply suggests that, after successful engagement, downstream adaptive mechanisms become progressively more important in determining the final phenotype.

Thus, target engagement and biological responsiveness represent related but distinct concepts.

Considering endogenous BPIFB4 induction

Investigator

If endogenous BPIFB4 contributes to the measured signal, could gene delivery itself stimulate native BPIFB4 expression?

AI

This possibility is biologically plausible.

The observed increase in total BPIFB4 protein may reflect not only production of the human transgene but also activation of endogenous regulatory mechanisms.

Such amplification would be consistent with a feed-forward adaptive response in which the therapeutic intervention enhances intrinsic resilience pathways.

However, the present dataset cannot experimentally distinguish endogenous induction from transgene-derived protein.

Consequently, this possibility should be presented explicitly as a hypothesis requiring validation using species-specific analytical methods.

Investigator

Therefore, the novelty lies not in the antibody itself, but in asking a different question.

AI

Exactly.

The methodological limitation was already recognized.

The analytical innovation arises from exploring its biological implications.

Implications for translational development

Investigator

Could this reinterpretation influence future therapeutic development?

AI

Potentially.

If biological pathway engagement proves more informative than transgene abundance alone, future translational studies may benefit from distinguishing:

- vector delivery,
- pathway activation,
- downstream adaptive response,

rather than considering them equivalent measures of therapeutic success.

This perspective may also encourage development of biomarkers reflecting biological pathway activation instead of relying exclusively on transgene expression.

Nevertheless, these implications remain conceptual and require prospective validation.

Investigator

Would you therefore recommend modifying the Discussion rather than the Results?

AI

Yes.

The experimental observations themselves remain unchanged.

The reinterpretation concerns their biological implications.

Consequently, these concepts are more appropriately discussed within the Discussion, where they can be clearly distinguished from experimentally demonstrated findings.

Emergence of a broader conceptual principle

Investigator

It seems that this discussion extends beyond BPIFB4.

AI

I agree.

The dialogue suggests a more general conceptual distinction applicable to many biological interventions.

Successful therapeutic delivery should not automatically be equated with biological responsiveness.

Rather, effective treatment may involve sequential levels of organisation:

Therapeutic intervention

↓

Target engagement

↓

Activation of adaptive biological pathways

↓

Structural remodelling

↓

Functional improvement

The BPIFB4 dataset provides one example in which this distinction emerged through structured reinterpretation.

Future studies will determine whether this organisational principle has broader applicability.

Editorial note

This dialogue represented one of the clearest examples of AI-assisted conceptual reinterpretation during the project. The methodological observation regarding antibody specificity was already known and reported in the original publication. The novelty emerged from asking a new biological question concerning the implications of that observation for interpreting therapeutic response. The resulting distinction between target engagement and adaptive pathway activation subsequently influenced the revised Discussion, translational perspectives, and future research roadmap, while remaining fully consistent with the published experimental findings.

Chapter 6. Reflection on AI-assisted scientific reasoning: from biological interpretation to methodological framework

Does AI actually "reason"?

Investigator

Throughout this dialogue I have the impression that AI is not simply retrieving information but reorganizing concepts.

Can this process legitimately be described as reasoning?

AI

The term "reasoning" should be used cautiously.

The AI does not reason in the human cognitive sense, nor does it possess scientific intuition or independent understanding of biological systems.

However, it can generate coherent analytical structures by integrating information provided during the dialogue with established scientific knowledge.

Within this project, the important point is not whether AI reasons exactly as humans do, but whether the dialogue supports investigator-led analytical exploration.

The scientific reasoning therefore emerges from the interaction rather than from either participant independently.

Investigator

So the unit of analysis becomes the dialogue itself rather than the AI.

AI

Exactly.

Neither the investigator nor the AI alone produced the final conceptual framework.

The framework progressively emerged through iterative questioning, critical evaluation, reformulation, and biological validation.

The dialogue therefore represents the analytical process.

Human and AI biases

Investigator

Humans have conceptual biases because we tend to interpret data according to our previous hypotheses.

Does AI exhibit different biases?

AI

Yes.

The sources of bias differ.

Human investigators often show:

- confirmation bias,
- attachment to previous hypotheses,
- disciplinary preferences,
- translational expectations.

AI, in contrast, may exhibit:

- preference for internally coherent explanations,
- tendency to integrate rather than discriminate,
- excessive confidence if not challenged,
- dependence upon the framing of the prompt.

Neither source of bias is inherently superior.

The analytical advantage arises when each compensates for the limitations of the other.

Investigator

Therefore the dialogue itself becomes a mechanism for exposing hidden assumptions.

AI

Precisely.

Repeated questioning forces both participants to justify conceptual choices.

Alternative interpretations become explicit instead of remaining implicit.

This transparency represents one of the principal methodological strengths of structured dialogue.

Hidden conceptual forks

Investigator

During our discussions I repeatedly noticed that the dialogue generated alternative conceptual pathways that had not initially occurred to me.

Could these represent hidden conceptual forks?

AI

Yes.

Scientific interpretation often follows the first biologically plausible hierarchy identified by investigators.

However, multiple compatible organizations may exist.

Structured dialogue encourages exploration of these latent alternatives before selecting the framework ultimately incorporated into the manuscript.

Importantly, many of these alternative paths are subsequently rejected.

Nevertheless, documenting their existence makes the analytical process more transparent.

Investigator

Therefore disagreement becomes informative.

AI

Yes.

Moments of disagreement frequently identify conceptual decision points.

Rather than representing failure of the dialogue, these forks often stimulate deeper analysis and lead to more robust final interpretations.

When should AI be challenged?

Investigator

Several times I asked you to moderate your conclusions because they appeared stronger than the available evidence.

Should this be considered an essential component of the methodology?

AI

Absolutely.

The dialogue should not consist of passive acceptance of AI outputs.

Investigator criticism constitutes an essential validation step.

Throughout this project numerous responses were modified after concerns regarding:

- excessive mechanistic certainty,
- overstatement of causality,
- unsupported translational implications,
- inappropriate terminology,
- distinction between observation and hypothesis.

These revisions illustrate investigator control rather than AI autonomy.

Emergence of the methodological contribution

Investigator

Initially I considered this a biological reinterpretation.

It now appears increasingly to be a methodological study.

AI

The dialogue evolved in exactly that direction.

The biological reinterpretation provided the case study.

The principal contribution gradually became the analytical methodology itself.

The study therefore shifted from asking:

"Can BPIFB4 be interpreted differently?"

toward:

"Can structured human–AI dialogue become a transparent analytical methodology for post-publication reinterpretation?"

Investigator

This was not the original objective.

AI

Correct.

The methodological contribution emerged progressively during the dialogue itself.

This illustrates one of the distinctive characteristics of exploratory analytical interaction: the objective may evolve as conceptual understanding develops.

Defining the role of AI

Investigator

One concern is that readers may conclude that AI generated the science.

How should this misunderstanding be avoided?

AI

The dialogue demonstrates the opposite.

The investigator:

- selected the dataset,
- formulated every scientific question,
- challenged interpretations,
- rejected unsupported conclusions,
- decided what entered the manuscript.

The AI:

- proposed alternative conceptual organizations,
- generated comparative frameworks,
- identified relationships among observations,
- suggested additional hypotheses,
- supported iterative refinement.

Scientific responsibility therefore remained entirely with the investigators.

Editorial note

This phase of the dialogue marked an important transition in the project. The emphasis progressively shifted from reinterpreting a specific biological dataset to defining a general methodology for investigator-led analytical dialogue with artificial intelligence. Many concepts subsequently incorporated into the revised Introduction, Methods, Discussion, and Conclusion—including the distinction between observed findings,

conceptual synthesis, and future hypotheses—originated during this methodological reflection rather than from the biological reinterpretation itself.

Chapter 7. Translating analytical dialogue into a scientific manuscript

From exploratory reasoning to scientific reporting

Investigator

At this stage, the dialogue has generated several conceptual frameworks. However, not all of them can be incorporated into the manuscript.

How should we decide what remains?

AI

The manuscript should not attempt to reproduce the entire analytical exploration.

Instead, it should retain only those concepts that satisfy four criteria:

- they remain fully supported by the published observations;
- they provide additional explanatory value;
- they are biologically plausible;
- they are clearly distinguished from experimentally demonstrated findings.

Ideas that remain speculative should either be removed or explicitly presented as hypotheses for future investigation.

Investigator

Therefore, the manuscript should become more conservative than the dialogue itself.

AI

Yes.

Exploratory dialogue intentionally generates multiple conceptual possibilities.

Scientific reporting requires progressive refinement.

Consequently, the final manuscript should represent the most biologically robust subset of the analytical discussion rather than the complete conversation.

Moderating the language

Investigator

Several interpretations sound stronger than the available evidence.

For example:

- resilience nodes,
- threshold engagement,
- vascular-first hierarchy,
- nuclear stress adaptation.

How should these concepts be presented?

AI

Throughout the manuscript, terminology should distinguish between:

Observed findings

and

Interpretative synthesis.

Expressions implying experimental demonstration should be replaced by language indicating conceptual interpretation.

For example,

instead of

"BPIFB4 functions as a resilience amplifier"

one may write

"The AI-assisted reinterpretation suggests that BPIFB4 may function as a resilience amplifier."

Similarly,

instead of

"vascular remodeling precedes functional recovery"

one may write

"vascular remodeling emerges as a candidate upstream adaptive layer."

This maintains scientific caution while preserving the conceptual contribution.

Investigator

This distinction should probably be applied consistently throughout the manuscript.

AI

Agreed.

The same principle should govern the Abstract, Results, Discussion, Figures, and Conclusion.

Separating observation from interpretation

Investigator

Is important to distinguish between data-supported observations and AI-generated interpretation.

How can this be achieved?

AI

The manuscript should consistently follow three sequential levels.

Level 1

Published experimental observations.

Level 2

Analytical reinterpretation generated through structured dialogue.

Level 3

Future hypotheses requiring prospective validation.

Maintaining this structure throughout the manuscript prevents conceptual overstatement and improves methodological transparency.

Investigator

This also seems applicable to the figures.

AI

Yes.

Figures should visually distinguish:

original experimental observations,

interpretative overlays,

and future conceptual implications.

This separation reinforces the methodological nature of the study.

Looking back at the dialogue

Investigator

After completing this analytical journey, what do you consider the principal contribution?

AI

The dialogue did not generate new experimental observations.

Neither did it establish new biological mechanisms.

Its principal contribution was to demonstrate that structured interaction between investigators and artificial intelligence can make explicit an analytical process that is usually performed informally and remains undocumented.

By recording the evolution of scientific reasoning—from the original hypothesis, through alternative conceptual pathways, to the final integrated framework—the dialogue itself becomes part of the scientific record.

Investigator

That was not the original intention when we started.

AI

No.

The initial objective was to reinterpret a published dataset.

During the dialogue, however, the methodological implications progressively became more important than the specific biological example.

Consequently, the project evolved naturally into a methodological proof-of-concept using BPIFB4 in progeria as its illustrative case study.

Final reflection

Investigator

Do you think this supplementary dialogue should be viewed simply as supporting material?

AI

Not entirely.

Although it accompanies the manuscript, it also serves as an example of transparent scientific reasoning.

Traditionally, scientific papers report only the final interpretation.

This document instead records how competing ideas were explored, refined, rejected, integrated, and ultimately translated into the published manuscript.

In doing so, it illustrates a possible framework for documenting structured investigator–AI analytical dialogue in future studies.

Editorial note

This final phase completed the transition from exploratory scientific dialogue to formal scientific reporting. The manuscript that emerged represents a filtered synthesis of the broader analytical exploration, retaining only concepts judged by the investigators to be compatible with the published evidence and sufficiently robust for scientific communication. The complete dialogue presented in this Supplement therefore documents not only the conclusions ultimately incorporated into the manuscript but also the reasoning process through which those conclusions were critically examined, refined, and contextualized.
